# Adoptive T-cell therapy of prostate cancer targeting the cancer stem cell antigen EpCAM

**DOI:** 10.1186/s12865-014-0064-x

**Published:** 2015-01-31

**Authors:** Zhenling Deng, Yanhong Wu, Wenbo Ma, Shuren Zhang, Yu-Qian Zhang

**Affiliations:** Department of Immunology, Cancer Hospital & Institute, Peking Union Medical College and Chinese Academy of Medical Sciences, Beijing, 100021 China

**Keywords:** Adoptive T-cell transfer, Chimeric antigen receptor, Cancer stem cell, EpCAM, Prostate cancer

## Abstract

**Background:**

Adoptive transfer of tumor infiltrating or circulating lymphocytes transduced with tumor antigen receptors has been examined in various clinical trials to treat human cancers. The tumor antigens targeted by transferred lymphocytes affects the efficacy of this therapeutic approach. Because cancer stem cells (CSCs) play an important role in tumor growth and metastasis, we hypothesized that adoptive transfer of T cells targeting a CSC antigen could result in dramatic anti-tumor effects.

**Results:**

An EpCAM-specific chimeric antigen receptor (CAR) was constructed to transduce human peripheral blood lymphocytes (PBLs) and thereby enable them to target the CSC marker EpCAM. To investigate the therapeutic capabilities of PBLs expressing EpCAM-specific CARs, we used two different tumor models, PC3, the human prostate cancer cell line, which has low expression levels of EpCAM, and PC3M, a highly metastatic clone of PC3 that has high expression levels of EpCAM. We demonstrate that CAR-expressing PBLs can kill PC3M tumor cells *in vitro* and *in vivo*. Despite the low expression of EpCAM on PC3 cells, CAR-expressing PBLs significantly inhibited tumor growth and prolonged mouse survival in a PC3 metastasis model, probably by targeting the highly proliferative and metastatic population of cancer cells.

**Conclusions:**

Our data demonstrate that PBLs expressing with EpCAM-specific CARs have significant anti-tumor activity against prostate cancer. Therefore, the adoptive transfer of T cells targeting EpCAM could have great potential as a cancer treatment.

## Background

Adoptive T-cell immunotherapy involves using *ex vivo* isolated and expanded autologous or allogeneic tumor-reactive lymphocytes to treat cancer patients. It has been highly effective in treating patients with metastatic melanoma and objective responses have been detected in 50% of patients [1,2].

Since tumor-infiltrating lymphocytes with tumor-specific receptors can only be generated from some cancer patients, adoptive T-cell therapy has been improved by introducing antigen receptors into circulating lymphocytes. To do this, genes encoding T-cell receptors isolated from high avidity, tumor-specific T cells or chimeric antigen receptors (CAR) containing an antibody-based external receptor structure and intracellular T-cell signaling domains, such as CD3ζ, are introduced into lymphocytes by retroviral or lentiviral vectors. Because CARs can induce T cells to attack tumors in an MHC-unrestricted manner, the application of adoptive T-cell therapy in cancer treatments has expanded. Currently, multiple clinical trials investigating CARs that recognize cell surface tumor antigens are underway, including for the treatment of lymphoma, chronic lymphocytic leukemia, melanoma, and neuroblastoma [3-5].

Cancer stem cells (CSCs) enable the tumor to grow and metastasize, therefore, eradicating CSCs is expected to provide cancer patients long-term disease-free survival. However, CSCs have also been demonstrated to be more resistant to chemotherapy and radiotherapy [6]. Currently, the research on immunotherapies targeting CSCs is limited.

In this study, we developed a new adoptive immunotherapy that targets cancer stem cell antigen, epithelial cell adhesion molecule (EpCAM). Studies have shown that EpCAM is expressed on CSCs from breast, colon, pancreas, and prostate tumors [7-11]. In breast cancer, EpCAM^+^ CD44^+^ CD24^−^ lineage^−^ cells are 10 times more likely to form tumors than the EpCAM^−^ CD44^+^ CD24^−^ lineage^−^ population [7]. In addition, our previous studies show that EpCAM^+^ cells of the human prostate cancer cell line PC3 display higher proliferation rates than EpCAM^−^ or unsorted PC3 cells. Interestingly, PC3M cells, a highly metastatic clone of PC3, express much higher levels of EpCAM than PC3, which suggests that EpCAM expression is associated with the proliferation and metastasis of prostate cancer cells.

In this paper, we show that human peripheral blood lymphocytes (PBLs) expressing EpCAM-specific CARs can kill PC3M cells *in vitro* and *in vivo*. Interestingly, despite the low expression level of EpCAM on PC3 cells, lymphocytes targeting EpCAM can cause significant killing and inhibit the metastasis of PC3 cells in NOD/SCID mice. This indicates that immunotherapies targeting CSCs, a small population of cancer cells, could result in distinct anti-tumor effects. Our results suggest that adoptive T-cell therapy targeting CSCs is a promising therapeutic strategy for cancer treatment.

## Results

**Figure 1:**
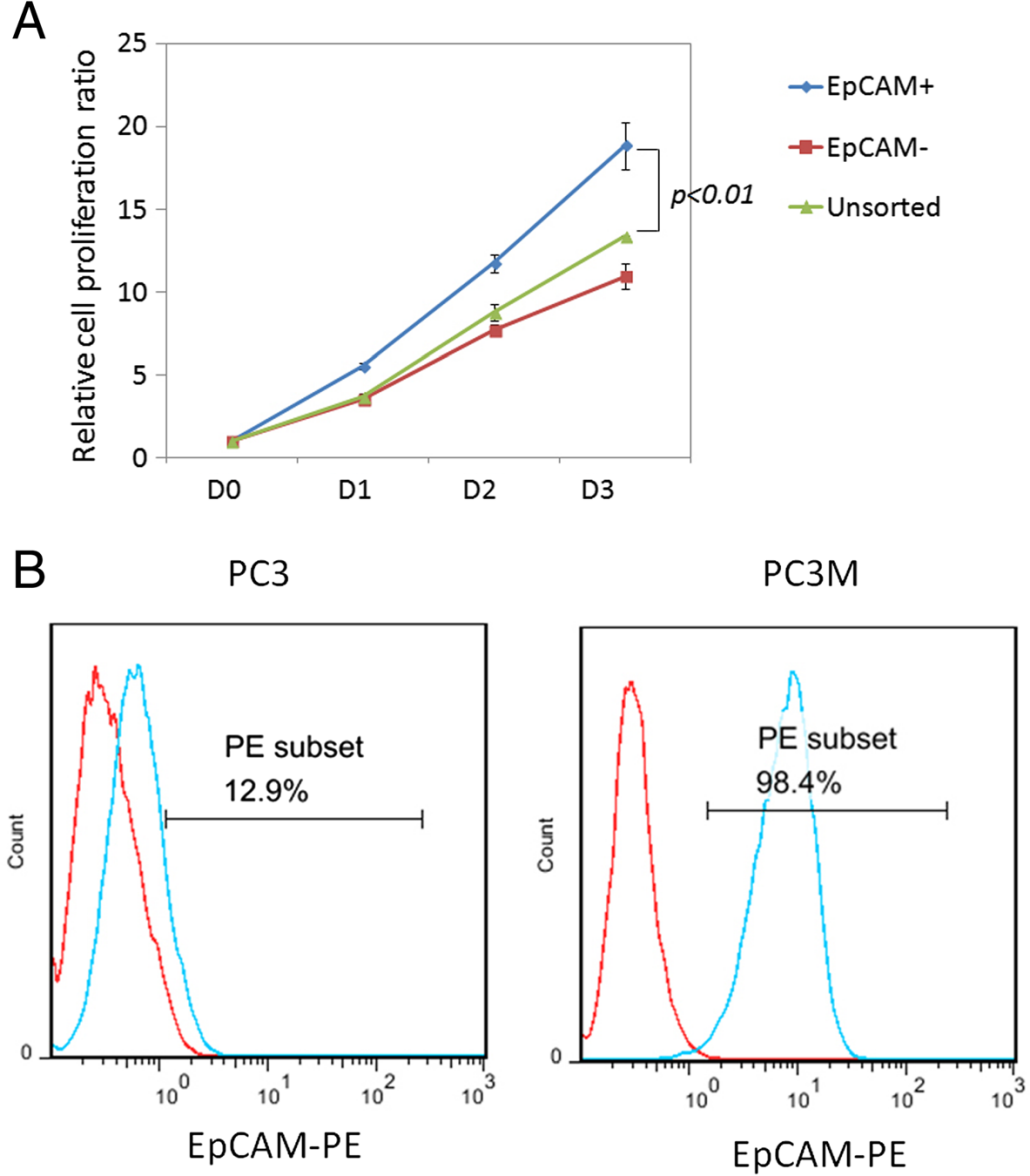
**EpCAM expression is associated with the proliferation and metastatic potential of PC3 cells. A)** The proliferation rates of EpCAM^+^, EpCAM^−^, and unsorted PC3 cells were determined with a CCK-8 assay. Statistical analysis was performed using a two-way ANOVA. **B)** The expression of EpCAM on PC3 and PC3M cells was determined by staining the cells with a PE-conjugated anti-EpCAM antibody.

Studies have shown that EpCAM is expressed on CSCs from prostate cancer and its expression is associated with prostate cancer cell proliferation, tumorigenesis, metastasis, and chemo/radioresistance [12,13]. To verify these findings, we sorted EpCAM^+^ and EpCAM^−^ cells from the PC3 cell line and performed a CCK-8 assay to examine the effect of EpCAM expression on PC3 cell proliferation. EpCAM^+^ cells displayed higher proliferation rates than EpCAM^−^ or unsorted cells (Figure 1A). We also compared the expression levels of EpCAM on PC3 and PC3M cells and found that EpCAM is expressed more on PC3M cells (12.9% *vs*. 98.4%, respectively; Figure 1B). This suggests that EpCAM is associated with the metastatic potential of prostate cancer cells.

**Figure 2:**
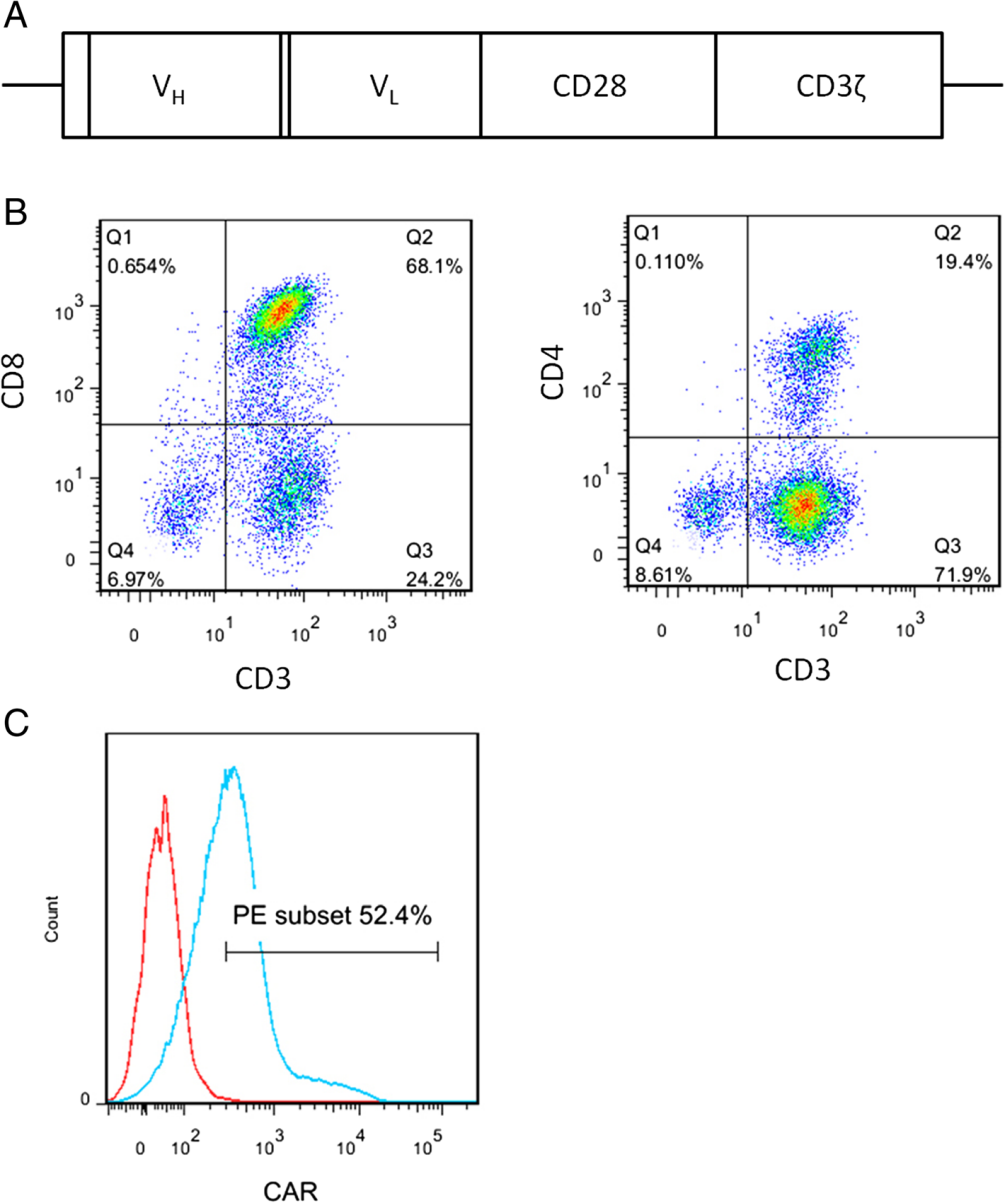
**Human PBLs transduced with retroviruses encoding the EpCAM-specific CAR. A)** A diagram of the EpCAM-specific CAR, including, V_H_, anti-human EpCAM immunoglobulin heavy chain variable region; V_L_, anti-human EpCAM immunoglobulin light chain variable region; CD28, consisting of part of the extracellular region and the entire transmembrane and intracellular regions of CD28, and CD3ζ, the cytoplasmic region only. **B)** PBLs were stimulated with OKT3 and anti-human CD28 antibodies for 2 d before being transduced with retroviruses encoding the EpCAM-specific CAR. Five days later, the PBL phenotypes were examined by staining with anti-CD3, anti-CD8, and anti-CD4 antibodies. **C)** Transduction efficiency was determined by staining with biotinylated protein L and PE conjugated streptavidin. PBLs not transduced were used as a negative control.

To target EpCAM, we designed an EpCAM specific CAR, which consists of an anti-EpCAM single chain variable fragment that was derived from the anti-human EpCAM mouse hybridoma clone C215 [14], a portion of human CD28, and the intracellular domain of human CD3ζ (Figure 2A). The CAR was ligated into the retroviral vector pLNCX. Retroviruses were produced using the retrovirus packaging kit, Ampho (Takara) and the 293 T packaging cell line. The retroviruses were used to transduce human PBLs that had been stimulated in culture with anti-CD3 and anti-CD28 for 2 d. The phenotype of the transduced PBLs was analyzed 5 d after retrovirus transduction by staining the cells with anti-CD3, anti-CD8, and anti-CD4 antibodies (Figure 2B). CD8 and CD4 were expressed by 68.1% and 19.4% of the transduced cells, respectively. To examine the transduction efficiency, we used protein L to detect the expression of CARs on transduced lymphocytes. Protein L is an immunoglobulin binding protein that can be used to determine CAR expression [15]. Five days after transduction, 54.2% of PBLs expressed the CAR (Figure 2C), which is similar to the transduction efficiency of PBLs reported previously [16].

**Figure 3:**
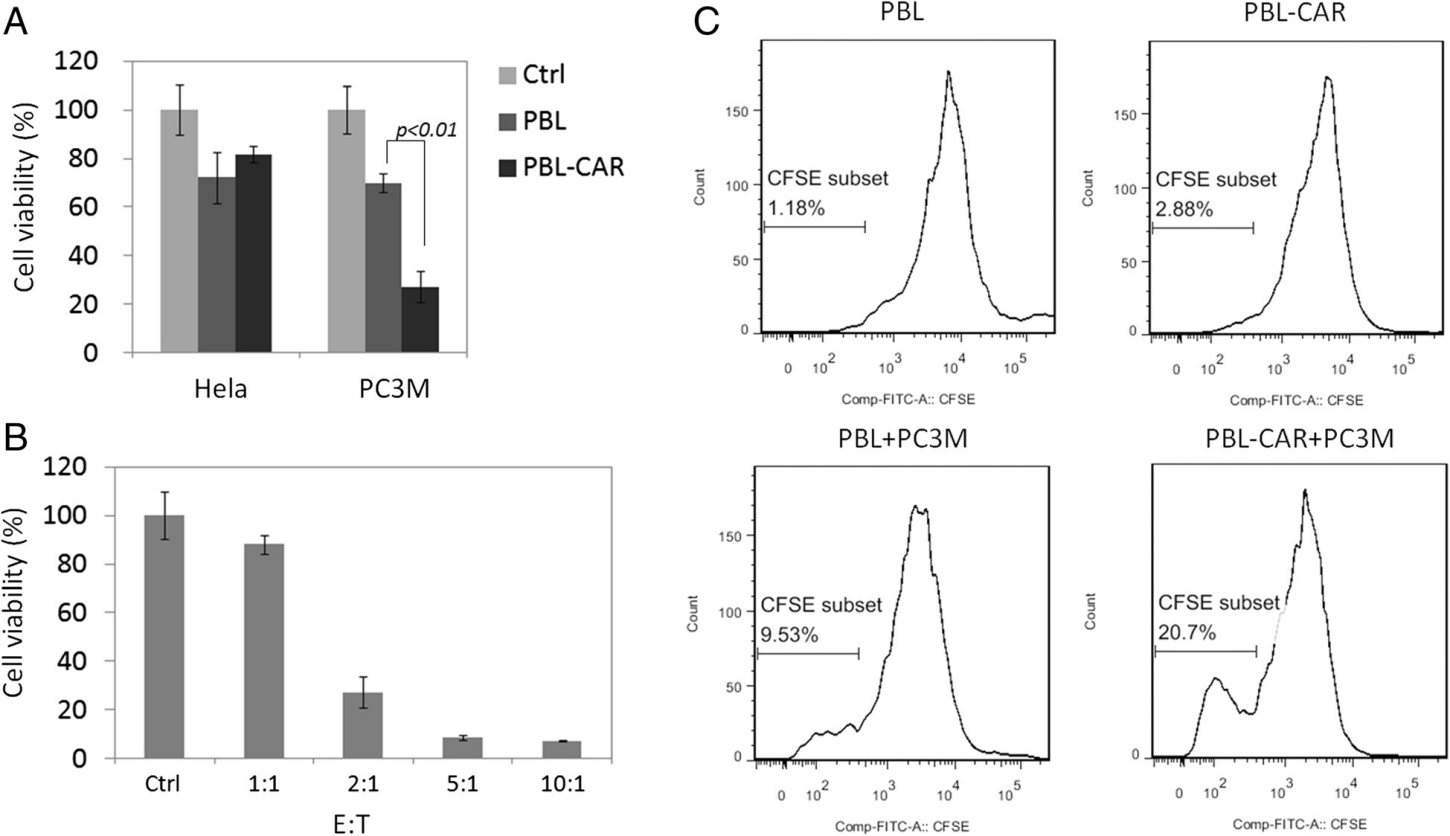
**Human PBLs transduced with the EpCAM-specific CAR cause cytotoxicity of PC3M tumor cells. A)** PBLs were stimulated with OKT3 and anti-human CD28 antibodies for 2 d before being transduced with retroviruses encoding the EpCAM-specific CAR or an empty vector. Five days later, transduced cells were incubated with Hela-luc or PC3M-luc tumor cells at an E:T ratio of 2:1. After 24 h of incubation, luciferin was added and the cell viability was examined by luminescence imaging. Tumor cells alone were used as a control. Statistical analysis was performed using a Student’s *t*-test. **B)** PBLs were stimulated as in **(A)** and were transduced with the EpCAM-specific CAR. Five days later, the transduced cells were incubated with PC3M-luc cells at different E:T ratios, including 1:1, 2:1, 5:1, and 10:1. After 24 h of incubation, luciferin was added and cell viability was determined by luminescence imaging. PC3M tumor cells alone were used as controls. **C)** PBLs were stimulated as in **(A)** and transduced with the EpCAM-specific CAR or control vector. Five days later, the cells were labeled with CFSE and were co-cultured with or without PC3M cells at an E:T ratio of 2:1. Three days later, the cells were collected and stained with the anti-CD8 antibody and cell proliferation was examined by flow cytometry.

To investigate if PBLs transduced with the EpCAM-specific CAR can specifically target EpCAM^+^ cells, we compared the cell killing capabilities of PBLs on two different, luciferase-expressing tumor cell lines, PC3M with high expression of EpCAM and Hela cells that do not express EpCAM. The tumor cells were incubated with PBLs transduced with either the EpCAM specific CAR or control retroviruses. Luminescence imaging was conducted 24 h after incubation to examine the cancer cell viability and the results were compared with the luminescence intensity of tumor cells cultured alone. The luminescence was significantly reduced when PC3M cells were cultured with PBLs transduced with the EpCAM-specific CAR, when compared with the culture with PBLs transduced with control retroviruses (Figure 3A). No significant difference in luminescence was detected when either PBL was cultured with Hela cells. This indicates that PBLs transduced with the EpCAM-specific CAR can target and kill EpCAM^+^ tumor cells. A dose response was also identified for the killing of PC3M cells with PBLs expressing EpCAM-specific CARs, with the cytotoxicity detected correlating with the E:T ratio (Figure 3B). Consistent with the tumor cell killing, PBLs expressing EpCAM-specific CARs proliferated when co-cultured with PC3M cells (Figure 3C), which suggests that the CAR-expressing PBLs can be activated by PC3M cells.

**Figure 4:**
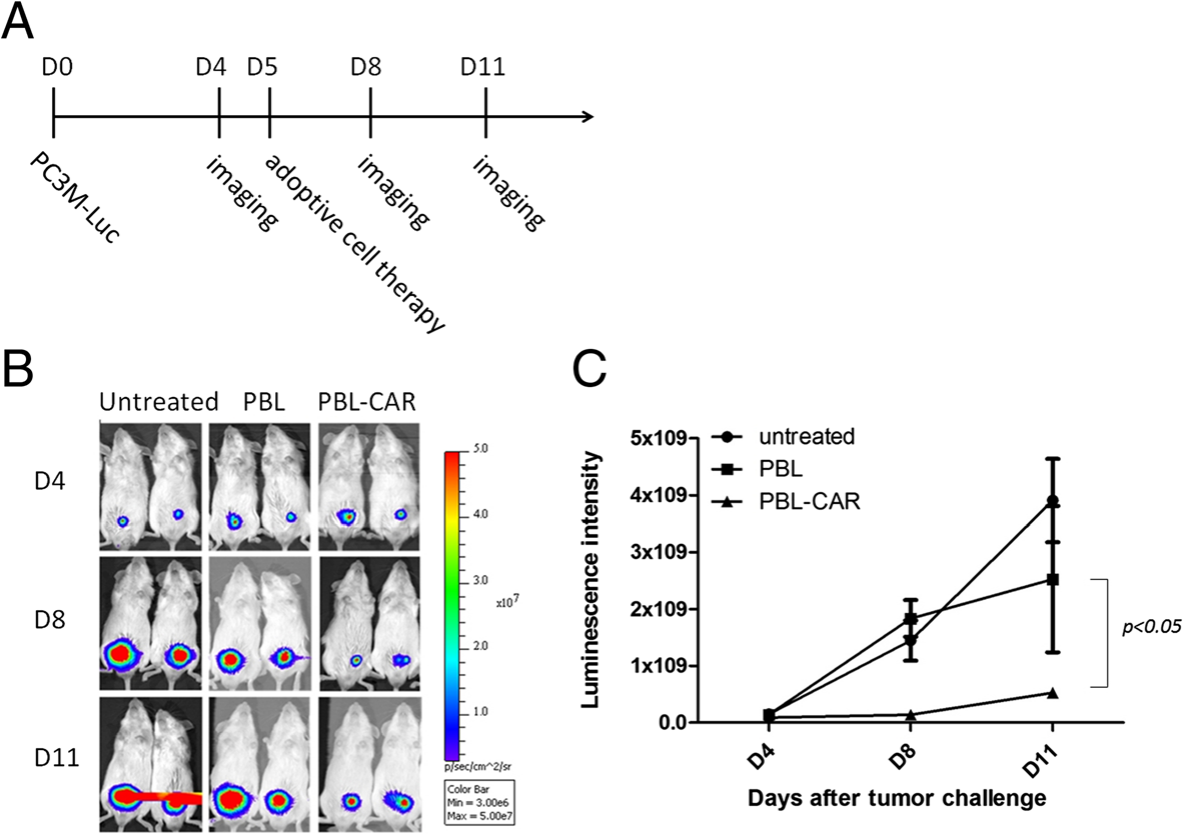
**Human PBLs transduced with the EpCAM specific CAR inhibit PC3M tumor growth** ***in vivo*** **. A)** A diagram of the experimental design. NOD/SCID mice were injected intraperitoneally with 5 × 10^5^ PC3M-luc cells on day 0 and tumor growth was examined by luminescence imaging on day 4. On day 5, PBLs transduced with the EpCAM-specific CAR or control vector were injected intravenously at 1 × 10^7^ cells/mouse and the anti-tumor effects were determined by luminescence imaging on days 8 and 11. **B)** Representative luminescence images of PC3M-luc tumor-bearing mice before and after PBL treatments. Tumor-bearing mice without treatment were used as controls. **C)** Quantitative results of the luminescence intensity of PC3M-luc tumor-bearing mice before and after PBL treatment. Statistical analysis was performed using a two-way analysis of variance.

To examine the tumor-killing abilities of CAR-expressing PBLs *in vivo*, we injected PC3M-luc tumor cells into NOD/SCID mice intraperitoneally. Five days later, PBLs transduced with the EpCAM-specific CAR or control retroviruses were injected intravenously. Luminescence imaging was conducted at different time points to analyze the tumor burden within the mice. PBLs expressing EpCAM-specific CARs significantly reduced PC3M tumor growth compared with PBLs transduced with control retroviruses, which showed no significant difference from the untreated tumor-challenged mice (Figure 4B and C).

**Figure 5:**
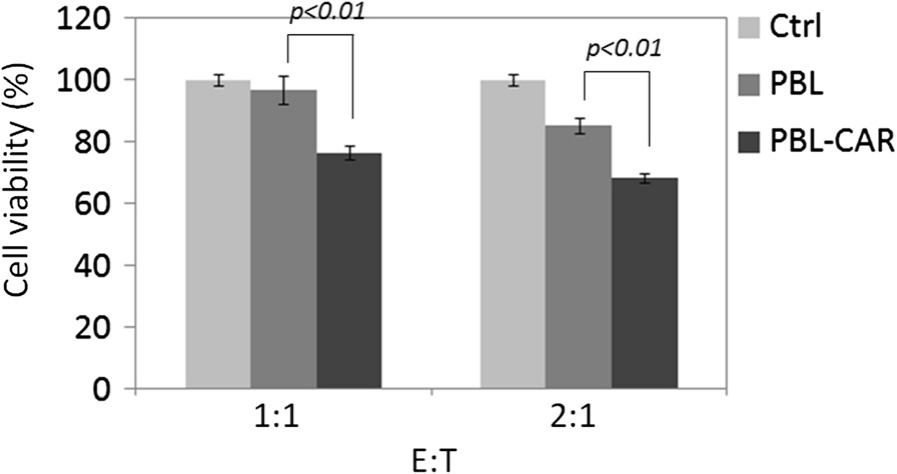
**Human PBLs transduced with EpCAM-specific CARs kill PC3 cells.** PBLs transduced with the EpCAM-specific CAR or control vector were incubated with PC3-luc cells at E:T ratios of 1:1 or 2:1. Luciferin was added 24 h later and cell viability was determined by luminescence imaging. PC3-luc cells incubated alone were used as controls. Statistical analysis was performed using a Student’s *t*-test.

Having found that PBLs expressing EpCAM-specific CARs can kill PC3M tumor cells *in vitro* and *in vivo*, we next aimed to determine if adoptively transferring PBLs expressing EpCAM-specific CARs can target CSCs. For this purpose, we used PC3 as the tumor model, of which only a small population of cells express EpCAM. Luciferase-expressing PC3 cells were incubated with EpCAM-specific or control PBLs at different E:T ratios. Luminescence imaging was performed to examine the cell viability 24 h later. Luminescence detected from the culture was significantly reduced when the tumor cells were cultured with CAR-expressing PBLs, whereas PBLs transduced with control retroviruses did not significantly reduce the luminescence (Figure 5). This indicates that PBLs targeting EpCAM are able to perform significant cytotoxic activities against PC3 cells and thus may be an effective therapeutic approach for prostate cancer.

**Figure 6:**
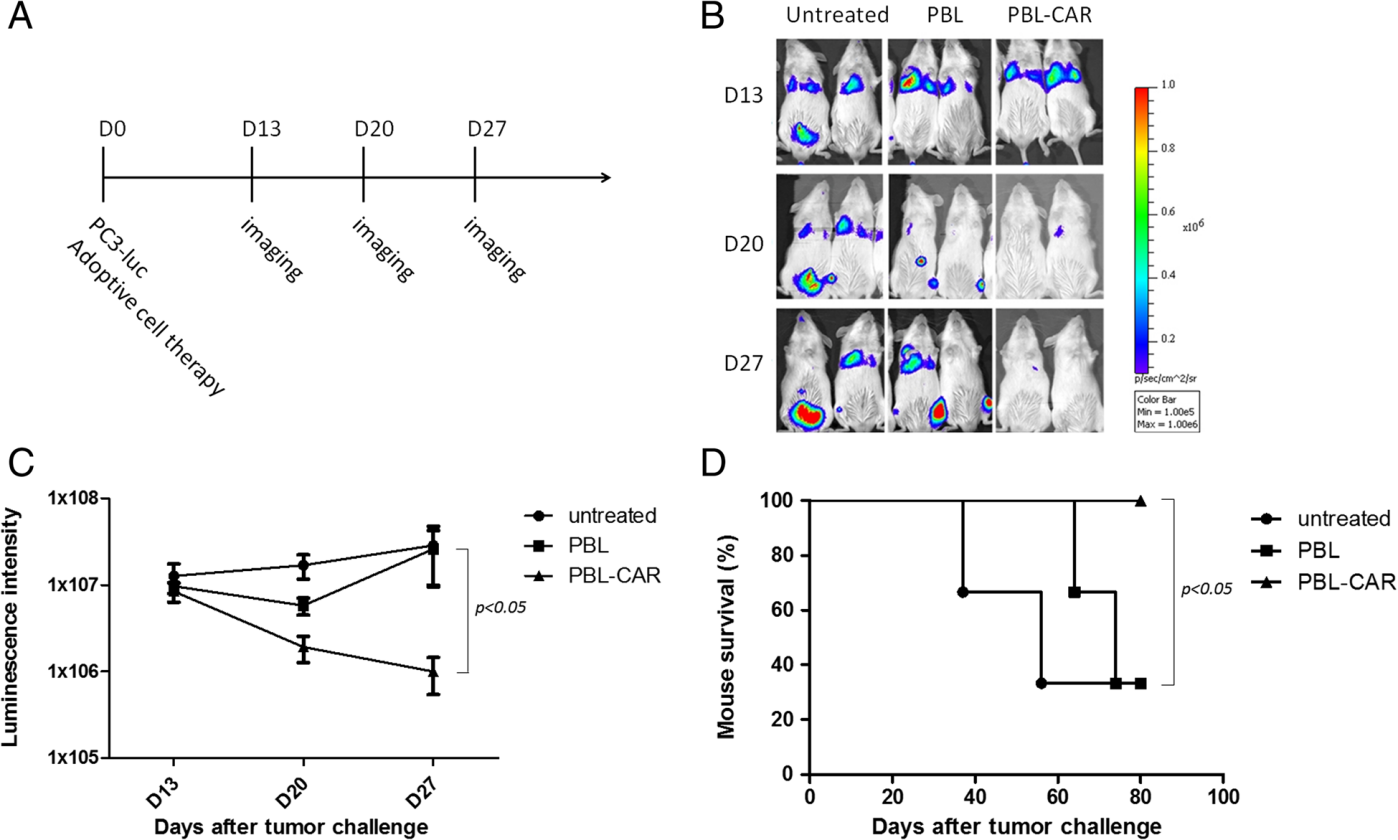
**Human PBLs transduced with the EpCAM-specific CAR inhibit PC3 tumor metastasis** ***in vivo*** **. A)** A diagram is displayed of the experimental model. NOD/SCID mice were injected intravenously with 5 × 10^6^ PC3-luc cells on day 0, followed 6 h later by intravenous injection of 5 × 10^6^ PBLs transduced with either the EpCAM-specific CAR or a control vector. Mice without treatment were used as controls. Anti-tumor activity was determined by luminescence imaging on days 13, 20, and 27. **B)** Representative luminescence images of PC3-luc tumor-bearing mice with or without PBL treatment. **C)** Quantitative results of the PC3-luc luminescence intensity from tumor-bearing mice treated with or without PBLs. Statistical analysis was performed using a two-way analysis of variance. **D)** Kaplan–Meier survival analysis of PC3-luc tumor-bearing mice with different PBL treatments. A comparison of the survival curves was made using a Log-rank test.

To test this hypothesis, we used a previously established PC3 metastasis model. PC3-luc tumor cells were injected into the tail vein of NOD/SCID mice and 6 h later PBLs transduced with EpCAM-specific CARs or control retroviruses were administered intravenously. Luminescence imaging was conducted at different times to examine the effect of PBL transfer on PC3 metastasis *in vivo*. Tumor metastasis was detected in the lung and bone of mice treated with PBLs transduced with control vectors 27 d after tumor challenge, whereas almost no luminescence was detected in mice treated with CAR-expressing PBLs (Figure 6B). Quantitative analysis of luminescence intensity also showed a significant difference between the group treated with CAR-expressing PBLs and the untreated group (Figure 6C). In addition, the PC3-bearing mice treated with CAR-expressing PBLs demonstrated prolonged survival compared with mice treated with control PBLs or untreated mice (Figure 6D). All mice treated with CAR-expressing PBLs were alive 80 days after tumor challenge, whereas only 1/3 of the mice in the two control groups survived. These data indicate that PBLs targeting EpCAM, the cancer stem cell antigen expressed on a small population of tumor cells, can perform significant anti-tumor effects.

## Discussion

In this study, we constructed a CAR targeting the cancer stem cell marker EpCAM and demonstrated that human PBLs transduced with the EpCAM-specific CAR can kill the prostate cancer cells PC3M and PC3, both *in vitro* and *in vivo*. Numerous clinical trials have been performed and are underway to examine the treatment of cancer with the adoptive transfer of tumor-reactive T cells; however, few studies have investigated the therapeutic potential of adoptive T-cell immunotherapy targeting CSCs. EpCAM is a marker that has been detected on CSCs from prostate cancer. In our study, we showed that EpCAM^+^ PC3 cells have higher proliferation rates than EpCAM^−^ cells. In addition, a more metastatic clone of PC3, PC3M, expresses higher levels of EpCAM (Figure 1A and B). This suggests that EpCAM expression is associated with the proliferation and metastatic potential of PC3 cells. Therefore, despite the low expression of EpCAM on PC3 cells, targeting EpCAM may cause dramatic tumor-killing effects.

PBLs transduced with retroviruses encoding EpCAM-specific CARs displayed significantly increased cytotoxicity against PC3M cells when compared with PBLs transduced with control retroviruses (Figure 3A). However, control PBLs also displayed significant cytotoxic activity against tumor cells relative to the untreated group. This is probably caused by direct recognition between alloreactive T cells from the healthy donors and human leukocyte antigen expressed by tumor cells [17]. The recognition between control PBLs and tumor cells is also reflected by the proliferation assay (Figure 3C), where cell proliferation was detected for the control PBLs co-cultured with PC3M cells.

At an E:T ratio of 2:1, PBLs expressing EpCAM-specific CARs lysed 73% of PC3M cells, whereas only 32% of PC3 cells were lysed (Figure 3A and Figure 5). The relatively low level of cytotoxicity detected for PC3 cells is probably because of its low expression of EpCAM. However, considering the association between EpCAM^+^ cells and PC3 cell proliferation and metastasis, targeting this small population of cells may cause dramatic tumor-killing effects. This is supported by the success of the CAR-expressing PBLs at protecting mice against PC3 development. Metastatic PC3 tumor cells were detected in the lung, peritoneal cavity, and bone of untreated mice and in mice treated with control PBLs, whereas treatment with EpCAM-specific PBLs significantly inhibited PC3 metastasis and prolonged mouse survival (Figure 6B-D).

The side effects of targeted therapies depend on the expression levels of the target in other tissues. Like other CSC antigens, EpCAM expression is not restricted to CSCs. As a cell adhesion molecule, EpCAM is expressed in a variety of epithelial tissues, which raises the concern that targeting EpCAM may lead to toxicity. Toxicity has been observed in several CAR trials, such as when targeting Her2/neu in colorectal cancer [18], or CD19 in B-cell malignancies [19]. One possible way to reduce acute toxicity by CAR transuded T cells is to administer multiple small doses of T cells rather than one large dose. Another possibility is to insert suicide genes into the CAR, which enables the T cells to be deleted when severe toxicity is observed. It is possible that with a better understanding of EpCAM and its function in cancer cells and CSCs, a component of EpCAM signaling may be identified that will provide a better and more specific target for cancer therapy.

## Conclusion

Our data demonstrate that the adoptive transfer of human PBLs with CARs specific for EpCAM can cause PC3M tumor cell killing *in vitro* and *in vivo*. Despite the low expression of EpCAM on PC3 tumor cells, EpCAM-specific PBLs had significant anti-tumor activity against PC3, probably by targeting the CSCs of prostate cancer. Our data suggest that adoptive transfer of T cells targeting CSC antigens is a promising therapeutic approach for treating cancer.

## Methods

### Developing retroviruses encoding an EpCAM-specific CAR

The EpCAM-specific CAR construct is similar to the FMC63-28z CAR (Genebank identifier HM852952.1), except the anti-CD19, single-chain variable fragment sequence is replaced with an anti-EpCAM fragment (sequence corresponds to Genebank identifier AJ564232.1). The construct was synthesized and inserted into a pLNCX retroviral vector. Retroviruses encoding the EpCAM-specific CAR or an empty pLNCX vector for controls were generated using the retrovirus packaging kit, Ampho (Takara), and a 293 T packaging cell line, following the manufacturer’s protocol.

### PBL preparation and retrovirus transduction

For PBL preparation, donor blood was obtained from healthy volunteers with consent from the Institutional Review Board of the Cancer Institute, Chinese Academy of Medical Sciences, and written informed consent for participation in the study was obtained from participants. After centrifugation on Ficoll-Hypaque density gradients (Sigma-Aldrich), PBMCs were plated at 2 × 10^6^ cells/mL in cell culture for 2 h and the non-adherent cells were collected. The cells were then stimulated for 2 d on a non-tissue-culture-treated 24-well plate coated with 1 μg/mL OKT3 (Biolegend) at 1 × 10^6^ cells/mL and in the presence of 1 μg/mL of anti-human CD28 antibody (Biolegend). For retrovirus transduction, a 24-well plate was coated with RetroNectin (Takara) at 4°C overnight, according to the manufacturer’s protocol, and then blocked with 2% BSA at room temperature for 30 min. The plate was then loaded with retrovirus supernatants at 300 μL/well and incubated at 37°C for 6 h. Next, 1 × 10^6^ stimulated PBLs in 1 mL of medium were added to 1 mL of retrovirus supernatants before being transferred to the pre-coated wells and cultured at 37°C for 2 d. The cells were then transferred to a tissue-culture-treated plate at 1 × 10^6^ cells/mL and cultured in the presence of 100 U/mL of recombinant human IL-2.

### Cell lines

PC3, PC3M, Hela, and 293 T cells were obtained from ATCC and were maintained in culture with DMEM medium (Gibco) supplemented with 10% FBS. PBLs were cultured in RPMI (Gibco) supplemented with 10% FBS, 1× nonessential amino acid, L-glutamine, sodium pyruvate, penicillin-streptomycin, and 0.1% β-mercaptoethanol. To establish PC3-luc, PC3M-luc, and Hela-luc stable cell lines, the luciferase gene was cloned from pGL4.17-luc/Neo and inserted into pLNCX retroviral vectors; the retroviruses were prepared as described above. To transduce the tumor cell lines, retrovirus supernatants were mixed with cell culture medium at a ratio of 1:1, which was added to the tumor cells at 60–70% confluence with 15 μg/mL polybrene. Twenty-four hours after transduction, the cells were split into ten plates and 400 μg/mL of G418 was added to the culture. Culture medium was changed every 2–3 d, and 10–14 d later selected cells were passaged and maintained in culture medium supplemented with 200 μg/mL of G418.

### Animal models

Male NOD/SCID mice, 5–8 weeks of age, were purchased from Vital River Laboratories, and used in compliance with institutional animal healthcare regulations. For the PC3M *in vivo* model, 5 × 10^5^ PC3M-luc cells were intraperitoneally injected into mice and 5 d later 1 × 10^7^ PBLs transduced with the CAR or control vector were injected. For the PC3 metastasis model, PC3-luc cells were injected intravenously at 5 × 10^6^ cells/mouse and 6 h later 5 × 10^6^ PBLs transduced with the CAR or control vector were injected intravenously. Live animal imaging was performed as described previously [20], briefly, the mice were intraperitoneally injected with 15 μg/μL of luciferin (Promega) in 200 μL and 10 min later luminescence imaging was conducted with an IVIS system (Xenogen/Caliper Life Sciences). For the *in vivo* experiments, five mice were used per group and each experiment was repeated at least twice.

### CCK-8 assay

Sorted or unsorted PC3 cells in 100 μL of medium were seeded in a 96-well plate at 2,500 cells/well; control wells received 100 μL of medium only. Ten microliters of CCK-8 solution (Dojindo) was added to each well and after 4 h of incubation at 37°C, the cell number was determined by measuring the absorbance at 450 nm using a microplate reader. Cells were cultured for 24, 48, and 72 h and a CCK-8 assay was performed at each time point. The absorbance was subtracted with that of the control well and the resulting OD_450_ at each time point was divided by the starting value to calculate the relative proliferation ratio.

### Flow cytometry and cell sorting

PBLs were stained with FITC, PE, or Percp-Cy5.5 conjugated CD3, CD4, or CD8 antibodies (eBioscience). Fluorescence was measured using a FACS Calibur flow cytometer and was analyzed using Flowjo software. To detect CAR transduced cells, PBLs were stained with an optimal concentration of biotinylated protein L (GeneScript), followed by staining with PE conjugated streptavidin (eBioscience). A PE-conjugated anti-human EpCAM antibody (eBioscience) was used to stain the tumor cells PC3 and PC3M and a FACSAria II cell sorter was used to sort EpCAM^+^ and EpCAM^−^ cells.

### Cytotoxicity assay

Luciferase-expressing tumor cells were seeded in a 96-well plate at 1 × 10^5^ cells/well and PBLs transduced with retroviruses were added at different E:T ratios. After incubation at 37°C for 24 h, luciferin (Promega) was added at a final concentration of 0.3 mg/mL and cytotoxicity was determined by luminescence imaging.

### CFSE proliferation assay

Retrovirus-transduced PBLs were labeled with CFSE (Invitrogen) according to the manufacturer’s protocol and incubated with tumor cells at an E:T ratio of 2:1. Three days later, cells were collected and stained with Percp-Cy5.5 conjugated CD8 antibodies (eBioscience) and were analyzed by flow cytometry. The analysis was carried out on the CD8^+^ population.

### Statistical analysis

Data are presented as means ± standard error of the mean. To determine the significance of differences between samples or groups, a student’s *t*-test or two-way analysis of variance was used as indicated in the figure legends.
